# Ancient Herb, Modern Metabolomics: Primary and Secondary Metabolite Profiling of Wild Fennel (*Foeniculum vulgare* Mill.) Accessions Under Drought

**DOI:** 10.3390/plants15172652

**Published:** 2026-08-29

**Authors:** Anja Batel, Nikola Major, Marta Anđelini, Nina Išić, Tvrtko Karlo Kovačević, Dean Ban, Igor Pasković, Smiljana Goreta Ban

**Affiliations:** Department of Agriculture and Nutrition, Institute of Agriculture and Tourism, K. Hugues 8, 52440 Poreč, Croatia; abatel@iptpo.hr (A.B.); nikola@iptpo.hr (N.M.); tvrtko@iptpo.hr (T.K.K.); dean@iptpo.hr (D.B.); paskovic@iptpo.hr (I.P.)

**Keywords:** abiotic stress, metabolomics, oxidative stress, genetic diversity

## Abstract

Fennel (*Foeniculum vulgare* Mill.) is an aromatic species of culinary and medicinal value, but the metabolic basis of its drought response and the extent of variation among wild populations remain poorly characterized. We investigated eleven wild fennel accessions from the Mediterranean region of Croatia under control and drought conditions, combining morphological measurements with targeted profiling of 78 primary and 55 secondary metabolites. Drought significantly reduced biomass, leaf area, length, and width, while leaf dry matter content increased. Primary metabolism shifted markedly: amino acids accumulated, with asparagine and arginine increasing by over 20-fold, whereas TCA-cycle (tricarboxylic acid cycle) organic acids declined. Pathway analysis identified Alanine, aspartate, and glutamate metabolism as most strongly affected by drought treatment. A decline in the GSH/GSSG ratio indicated oxidative stress under water shortage. Among secondary metabolites, free hydroxycinnamic acids and flavonoid aglycones accumulated while most glycosylated flavonoids declined, and eight compounds showed genotype-dependent responses in drought conditions. These results reveal accession-specific metabolic adjustments to drought and characterize Croatian wild fennel as a genetic resource with distinct phytochemical profiles relevant to future conservation and breeding.

## 1. Introduction

Wild fennel (*Foeniculum vulgare* Mill.) is one of the oldest and most widespread aromatic plants of the Apiaceae family, with its use in human nutrition and traditional medicine dating back thousands of years. This perennial herb is native to the Mediterranean and has successfully naturalized across various regions worldwide, showing remarkable adaptability to different environmental conditions [1]. Despite its historical significance and broad distribution, wild fennel remains relatively understudied compared to other aromatic plants, particularly in terms of its physiological and biochemical responses to environmental stresses [2].

Drought stress is one of the most significant abiotic stresses impacting plant growth, productivity, and survival worldwide. As climate change accelerates, the frequency and severity of drought events are expected to rise, making it more important to understand how plants respond to drought [3]. Plants have evolved sophisticated mechanisms to cope with water scarcity, involving both morphological adaptations and metabolic adjustments. The metabolic aspect of drought response is particularly intricate, involving modifications in both primary metabolism–central pathways that support growth and maintenance, and secondary metabolism–specialized pathways that produce defense and signaling compounds [4]. The Mediterranean climate, characterized by hot, dry summers and mild, wet winters, exerts natural selection pressure for drought tolerance in native flora. Plants in these ecosystems have developed various strategies to withstand prolonged periods of water shortages, making Mediterranean species valuable models for understanding drought adaptation mechanisms [5].

Primary metabolites, including sugars, amino acids, organic acids, and other central metabolic compounds, play crucial roles in plant responses to drought. These compounds serve multiple functions during water stress, acting as osmolytes, energy sources, and precursors for protective compounds [6]. Carbohydrate metabolism undergoes substantial changes during drought stress, with plants typically accumulating soluble sugars such as sucrose, glucose, and fructose. These sugars function as osmotic adjustment compounds, helping to maintain cell turgor pressure under water-deficit conditions [7]. Amino acid metabolism is profoundly affected by drought stress, with proline accumulation among the most well-documented responses. Beyond proline, other amino acids, including gamma-aminobutyric acid (GABA), branched-chain amino acids, and aromatic amino acids, also accumulate during water stress [3].

Secondary metabolites, while not directly essential for basic cellular processes, play crucial roles in plant adaptation to environmental stresses. These specialized compounds include phenolic compounds, terpenoids, alkaloids, and other bioactive molecules that contribute to stress tolerance through various mechanisms, including antioxidant activity, UV protection, and antimicrobial effects [4,6]. Phenolic compounds, including phenolic acids, flavonoids, and other polyphenolic structures, typically increase during drought stress. These compounds serve as powerful antioxidants, helping plants cope with the oxidative stress that commonly accompanies water deficit [8].

Research on fennel responses to drought stress has revealed several important physiological and biochemical adaptations. Studies have demonstrated that fennel exhibits active osmoregulation mechanisms that help prevent sharp decreases in relative water content during water limitation. Fennel can sustain relatively high photosynthetic rates under mild stress conditions; however, severe drought significantly reduces these parameters [9]. Genetic variation in drought responsiveness among fennel genotypes has also been documented. Hosseini et al. [10] reported significant genotype-dependent differences in physiological drought responses among fennel populations, with mating systems further shaping the extent of this variability. Similarly, Askari and Ehsanzadeh [11] working with six fennel genotypes of different geographical origin, classified genotypes into drought-tolerant, moderately tolerant, and drought-sensitive groups based on a stress susceptibility index, and showed that tolerant genotypes exhibited greater accumulation of osmolytes (proline, soluble carbohydrates) and higher antioxidant enzyme activity (catalase, ascorbate peroxidase, superoxide dismutase) than sensitive genotypes under water deficit. These findings demonstrate that fennel populations harbor substantial, exploitable genetic variation in drought-response mechanisms.

Water limitation typically reduces plant height, umbel production, seed yield, and overall biomass, while simultaneously increasing essential oil concentration. These responses suggest a trade-off between growth and secondary metabolite production under resource-limited conditions, a pattern commonly observed in aromatic plants [12]. Comprehensive metabolomics analyses of fennel drought responses remain limited. Studies using different fennel populations and cultivars have revealed genetic variation in drought tolerance. This variation suggests that natural selection has produced diverse adaptive strategies within the species, making population-level studies particularly valuable for understanding the full range of fennel’s drought response capabilities [10].

Despite the ecological and economic importance of wild fennel, significant gaps remain in our understanding of its drought response mechanisms. Most previous studies have focused on morphological and physiological responses, with limited attention to the underlying metabolic changes that drive these responses. The few metabolomics studies conducted on aromatic plants under drought stress have revealed complex metabolic adjustments [13] but fennel-specific responses remain largely uncharacterized. Studying multiple accessions from the same geographical region provides opportunities to understand variability of drought responses within a species. Such approaches can reveal the metabolic basis of local adaptation and identify key metabolites associated with drought tolerance. This information is valuable for both fundamental understanding of plant stress biology and practical applications in crop breeding and conservation.

## 2. Results

### 2.1. Biomass Accumulation and Leaf Morphology Under Drought Stress

Eleven accessions of *Foeniculum vulgare* Mill. originating from Croatia were investigated in this study, with accession numbers and specific collection sites detailed in Table S1. Analysis of variance revealed significant effects of accession (Acc), treatment (Tr), and their interaction (Acc × Tr) on the measured morphological and physiological traits of 11 fennel accessions subjected to drought stress (Table 1). Leaf, stem, and total dry weight (DW), along with leaf area and length, showed a significant interaction effect between Acc and Tr. The main effects were significant for leaf width, as well as leaf, stem, and total dry matter (DM) percentage (except for the Acc effect on leaf DM). Specifically, the accession effect was significant for all measured parameters (*p* < 0.02) except for leaf DM (*p* = 0.49). The treatment effect was significant for all parameters (*p* < 0.001). Notably, the Acc × Tr interaction was significant for dry weight parameters, leaf area, and leaf length. Leaf dry matter parameters and leaf width showed no significant accession and treatment interaction.

**Figure 1:**
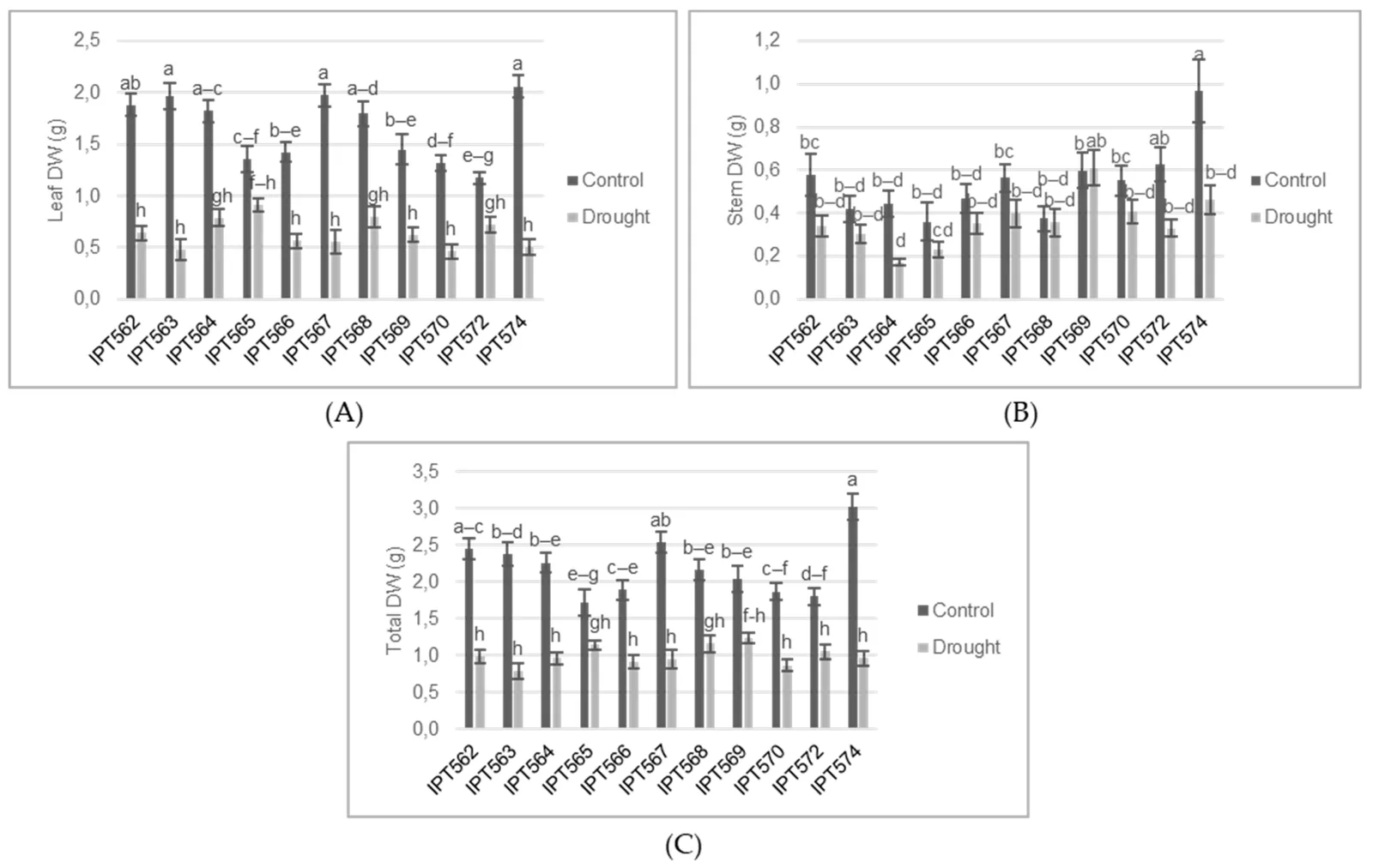
Parameters with significant main interaction effect; (**A**) Leaf dry weight, (**B**) Stem dry weight, (**C**) Total DW (sum of leaf and stem dry weight). Bars represent mean values ± standard error for each accession (*n* = 5). Different lowercase letters indicate statistically significant differences between mean values at *p* < 0.05 determined by a two-way ANOVA and Tukey’s test.

The impact of drought was more pronounced on the leaves and total DW than on the stems. Significantly lower total and leaf DW were observed under drought conditions compared to the control across all accessions (except IPT565). Significantly lower stem DW was observed under drought only for accession IPT574 (Figure 1A–C). Under drought conditions, no significant differences among the accessions in leaf, stem, and total DW were observed, except for significantly higher stem DW in IPT569 compared to IPT564 and IPT565 (Figure 1B). Under control (water-available conditions), accessions IPT563, IPT567, and IPT574 had significantly higher leaf DW than IPT565, IPT566, IPT569, IPT570, and IPT572 (Figure 1A), while IPT574 exhibited significantly higher stem DW compared to all other accessions (except for IPT572) (Figure 1B). In the control group, the total DW of IPT574 was comparable to that of IPT562 and IPT567 and significantly higher than that of all other accessions (Figure 1C).

**Figure 2:**
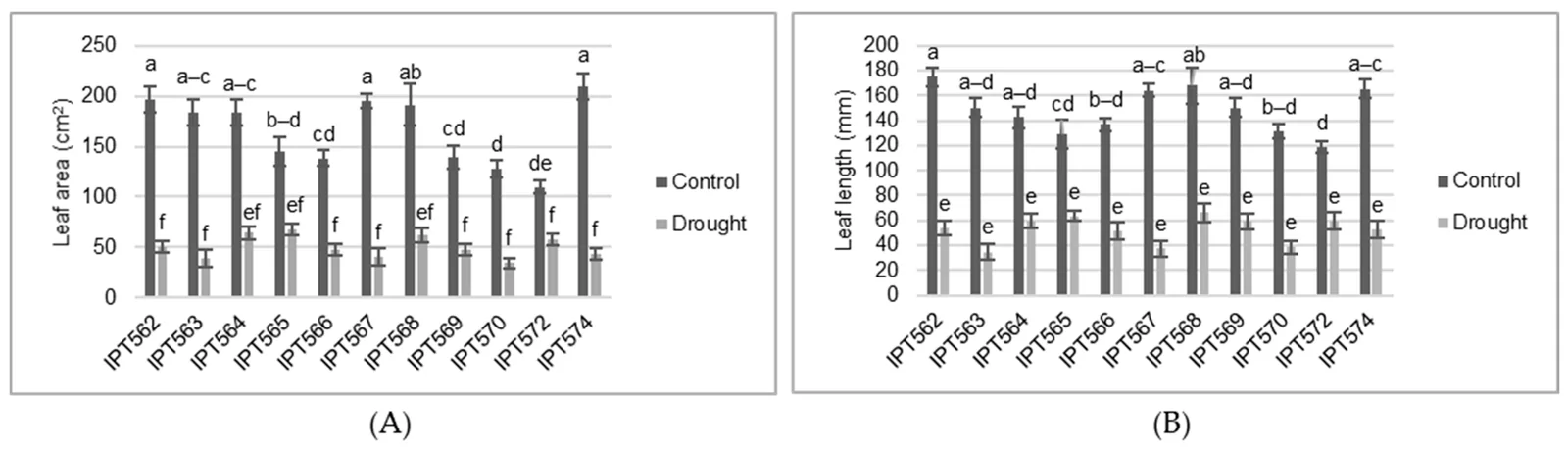
Parameters with significant main interaction effect: (**A**) Leaf area and (**B**) Leaf length. Bars represent mean values ± standard error for each accession (*n* = 5). Different lowercase letters indicate statistically significant differences between mean values at *p* < 0.05 determined by a two-way ANOVA and Tukey’s test.

Leaf, stem, and total dry matter (calculated as the ratio of dry to fresh weight) were higher in drought-treated plants than in control plants, with an average increase of 20% across all accessions (Table 2). While no significant difference in leaf DM was observed among accessions, IPT569 had significantly higher stem DM than IPT563, IPT564, and IPT568, and significantly higher total DM than IPT565 (Table 2). Drought significantly reduced leaf area, length, and width compared to the control across all accessions (Figure 2A,B and Table 2). The greatest reductions in leaf area and length under drought were observed for IPT563, IPT567, and IPT574, while IPT572 showed the smallest reduction (Figure 2A,B). Drought treatment significantly reduced mean leaf width from 1.05 ± 0.02 cm to 0.94 ± 0.02 cm, representing a 10.5% decrease (Table 2). There were no significant differences between accessions under drought conditions in leaf area and length (Figure 2A,B). Under control conditions, accessions IPT562, IPT567, and IPT574 had significantly larger leaf areas than IPT565, IPT566, IPT569, IPT570, and IPT572. Similarly, leaves of accession IPT562 were significantly longer than leaves of accessions IPT565, IPT566, IPT570, and IPT572. Leaf width varied significantly among fennel accessions, with accession IPT564 producing wider leaves (average 1.15 ± 0.04 cm) compared to most accessions, including IPT562, IPT566, IPT568, IPT569, IPT570, and IPT572, irrespective of water availability (Table 2).

### 2.2. Primary Metabolites Under Drought Stress

Of the 78 analyzed primary metabolites (Table S1), 63 showed statistically significant differences, with 3 exhibiting a significant interaction effect: methionine sulfoxide, allantoin, and succinic acid. Fifty-nine primary metabolites were divided into 7 major groups: phytohormones, organic acids, amino acids and their derivatives, vitamins, antioxidants, and non-hormonal signaling molecules. During drought conditions, the concentrations of all phytohormones increased compared to the control, with abscisic acid and indole-3-acetic acid displaying the largest increases. Abscisic acid, indole-3-acetic acid, 6-benzylaminopurine, and methyl jasmonate had statistically significant treatment effects. Drought treatment significantly decreased concentrations of 2-ketoglutaric acid, citric acid, shikimic acid, and succinic acid, with 2-ketoglutaric acid reduced by more than 20-fold. Twenty-two of twenty-four amino acids had a non-significant interaction and accession effect, and a significant treatment effect. They were upregulated during drought, with asparagine and arginine increased by over 20-fold. Five out of seven vitamins were affected by the treatment, although not in the same way; vitamins E and B6 dropped twofold in drought-treated plants, while vitamins D2 and C rose twofold. Vitamins B5, E, and C had a significant accession effect. During drought, oxidative stress increased, as indicated by increased oxidized glutathione concentration and a decreased reduced glutathione (GSH) to oxidized glutathione (GSSG) ratio. The interaction effect was significant for the GSH/GSSG ratio.

**Figure 3:**
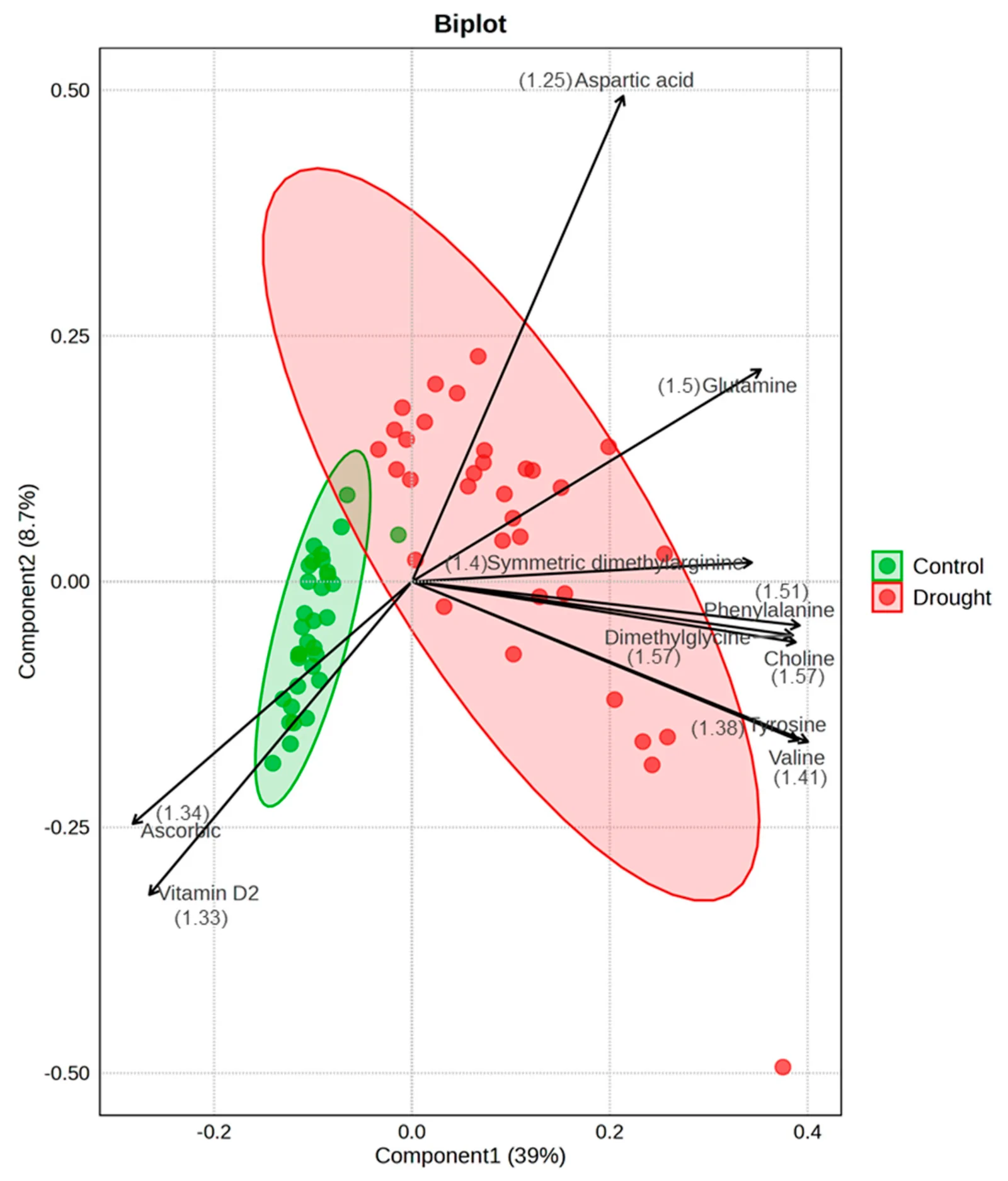
Partial Least Squares Discriminant Analysis (PLS-DA) biplot of primary metabolite profiles in 11 *Foeniculum vulgare* Mill. accessions under control and drought treatment. The analysis was performed with MetaboAnalyst. Each point represents a sample, with green dots indicating control and red dots indicating drought-treated plants. The separation of groups along the components reflects differences in metabolite composition in response to drought stress. Numbers in parentheses represent Variable Importance in Projection (VIP) scores of each metabolite.

Partial Least Squares Discriminant Analysis (PLS-DA) was conducted to examine the primary metabolite profiles of eleven *Foeniculum vulgare* Mill. accessions cultivated under control and drought conditions. The resulting biplot in Figure 3 displays a clear separation of samples by treatment, with distinct clusters representing control (green) and drought-treated (red) plants. Components 1 and 2 explained 39% and 8.7% of the total variance, respectively, indicating that these two main axes capture significant metabolic differences between the groups. Ellipses surrounding each group in the plot reflect consistent clustering, highlighting marked shifts in metabolite composition in response to drought. The Variable Importance in Projection (VIP) scores from the PLS-DA model are shown in parentheses next to each metabolite. Notably, dimethylglycine, choline, phenylalanine, and glutamine scored the highest VIP (>1.50), reflecting their prominent roles in distinguishing watering regimes.

**Figure 4:**
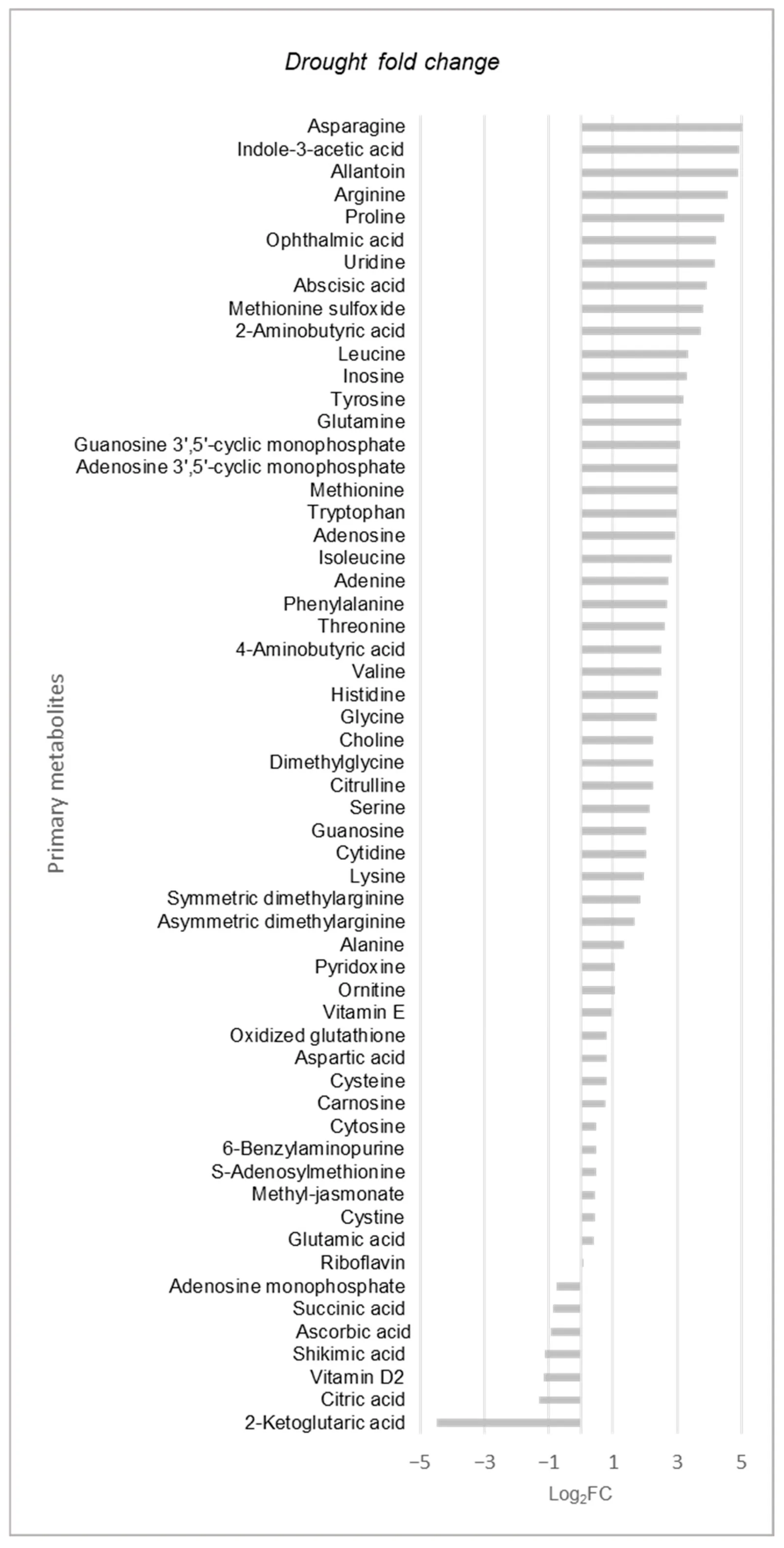
Drought-induced fold change in selected primary metabolite concentrations in leaves of 11 *Foeniculum vulgare* Mill. accessions. Primary metabolites were selected based on the statistical significance of the treatment effect (two-way ANOVA, post hoc Tukey, *p* < 0.05). Fold change was calculated as the log_2_ ratio of the mean concentration under drought to the mean concentration under control for each metabolite. Values > 0 indicate an increase, while values < 0 indicate a decrease in metabolite concentration under drought.

Figure 4 illustrates the changes in the concentrations of selected primary metabolites with a statistically significant treatment effect (*p* < 0.05, two-way ANOVA with post hoc Tukey) in the leaves of eleven *Foeniculum vulgare* Mill. accessions under drought conditions. The bar plot displays a log_2_ ratio of mean metabolite concentrations in drought-stressed versus control conditions. Among the analyzed metabolites, asparagine, indole-3-acetic acid, allantoin, arginine, and proline showed the most significant upregulation, with log_2_ fold changes ranging roughly from 4.5 to 5.0. In contrast, metabolites such as 2-ketoglutaric acid, citric acid, vitamin D2, and shikimic acid exhibited a decrease under drought conditions, with 2-ketoglutaric acid measured at over twenty-two times less under drought conditions compared to the control conditions. The extent of change caused by drought varied considerably among the metabolites, emphasizing a significant upregulation of primary metabolites.

**Figure 5:**
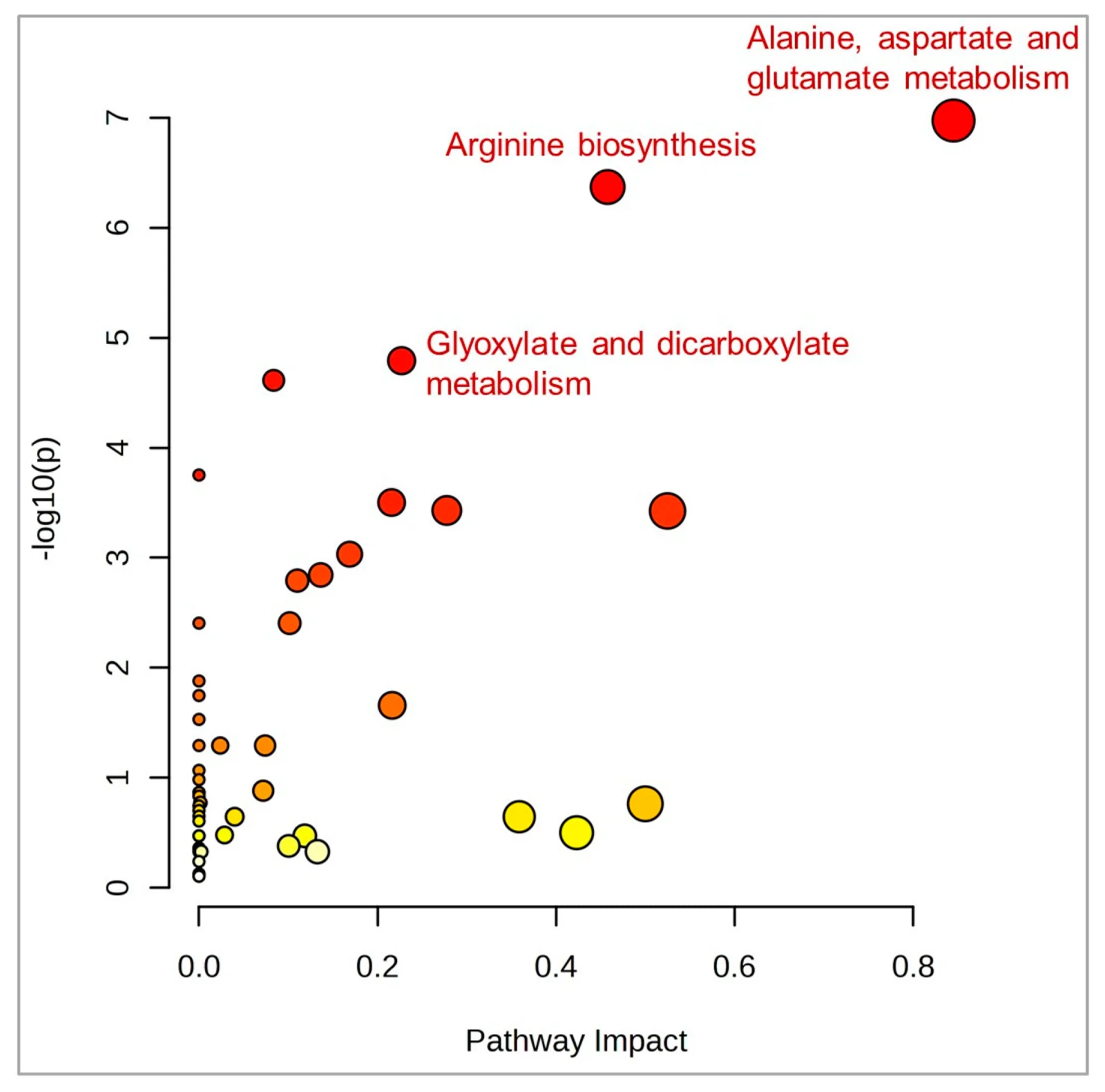
Pathway analysis overview of primary metabolites significantly increased under drought conditions in *Foeniculum vulgare* accessions, generated with *MetaboAnalyst*. Primary metabolites were selected based on statistical significance of treatment effect (two-way ANOVA, post hoc Tukey, *p* < 0.05). The *x*-axis represents the pathway impact value computed from pathway topological analysis, and the *y*-axis is the −log of the *p*-value obtained from pathway enrichment analysis. The size of each circle is proportional to the pathway impact, and the color reflects the degree of significance, ranging from yellow (lower −log(*p*)) to red (higher −log(*p*)). The pathways that were most significantly changed are characterized by both a high −log(*p*) value and a high impact value (top right region). Pathway identification details are provided in Table S2.

The pathway analysis of primary metabolites exhibiting statistically significant treatment effects performed using MetaboAnalyst is summarized in Figure 5 and detailed in Table S2. In total, 58 drought-responsive metabolites were mapped to different steps of 48 pathways. Thirteen pathways demonstrated notable enrichment, with an FDR value less than 0.05. The most affected pathway was Alanine, aspartate and glutamate metabolism, with the highest impact score (0.85) and the lowest FDR value (9.78 × 10^−6^), indicating strong involvement of amino acid metabolism in the drought response. Arginine biosynthesis and Glyoxylate and dicarboxylate metabolism were also highly ranked, combining high pathway impact with strong statistical significance. Other affected pathways included the One-carbon pool by folate, Cyanoamino acid metabolism, and Arginine and proline metabolism, highlighting both central carbon and nitrogen metabolic adjustments in response to water deficit. The data visualized in Figure 5 display these pathways by plotting impact against −log_10_(*p*-value), with the most pronounced changes in both statistical and biological significance highlighted accordingly. Figure S1 depicts Alanine, aspartate, and glutamate metabolism in detail, including metabolites that were detected in this study.

**Figure 6:**
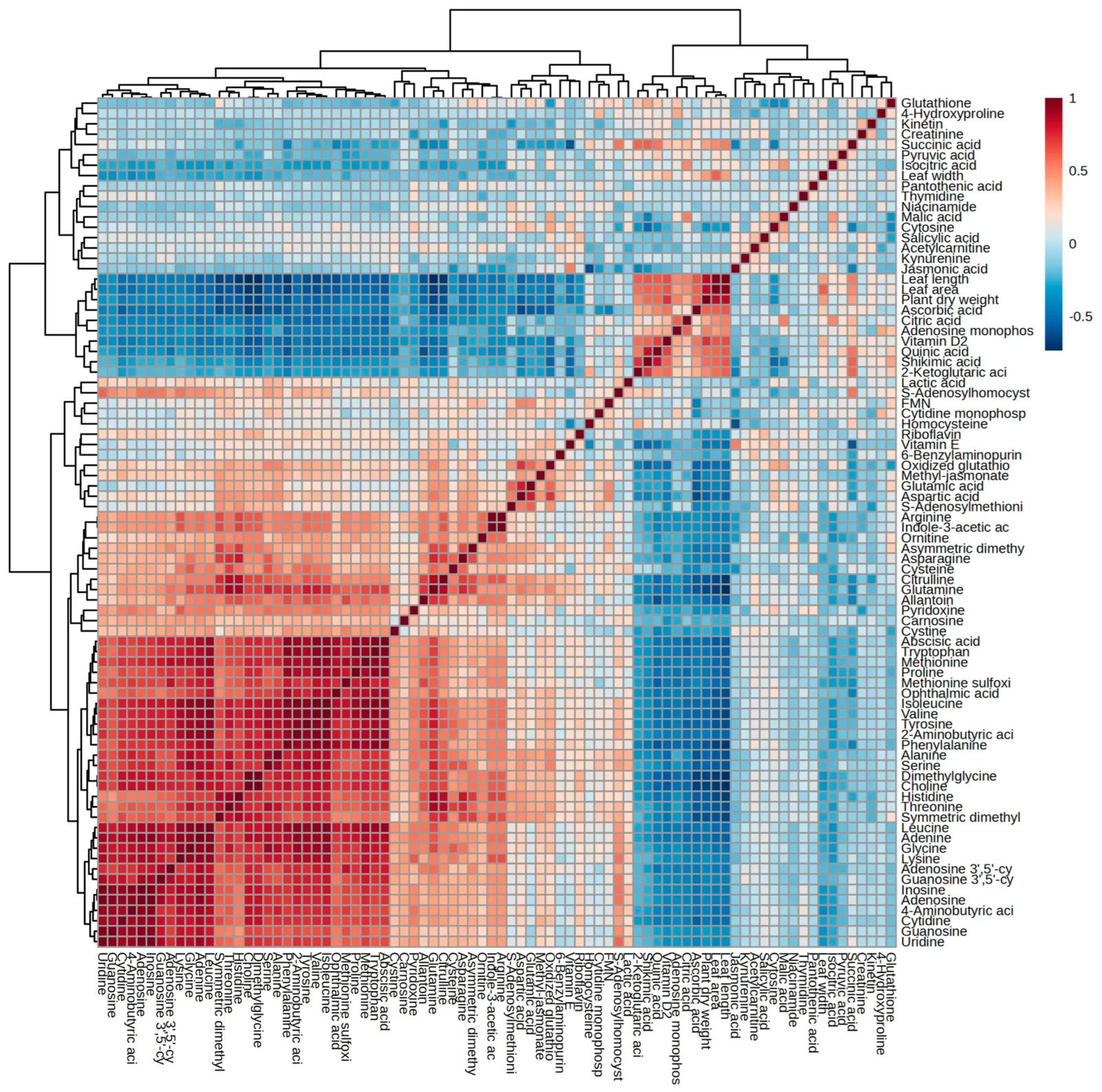
Heatmap of Pearson correlation coefficients between primary metabolite abundances and morphological parameters across all *Foeniculum* accessions. Colors indicate the direction and strength of correlations, with hierarchical clustering applied to traits and metabolites. Correlation analysis was performed in MetaboAnalyst.

A heatmap of Pearson correlation coefficients illustrating the pairwise relationships among a comprehensive set of primary metabolites alongside several morphological traits in *Foeniculum vulgare* Mill. is shown in Figure 6. The heatmap integrates metabolite concentrations with leaf morphological parameters, including leaf area, leaf length, leaf width, and total dry weight. Colors indicate the strength and direction of correlations: red shades representing positive correlations, blue shades indicating negative correlations, and white indicating no correlation. Hierarchical clustering based on correlation patterns groups metabolites and traits into two main clusters. The results demonstrate several noteworthy correlations between metabolites and leaf characteristics. Positive correlations were evident among metabolites involved in amino acid metabolism and nucleosides, which clustered together distinctly from morphological traits. Leaf length, leaf area, and plant dry weight formed a closely related cluster, positively correlating with ascorbic acid, citric acid, adenosine monophosphate, vitamin D2, quinic acid, shikimic acid, and 2-ketoglutaric acid. Conversely, certain metabolites, including dimethylglycine and choline, exhibited negative correlations with leaf morphological traits. Leaf width showed weaker and more variable correlations with metabolites compared to other leaf traits.

### 2.3. Secondary Metabolites Under Drought Stress

Among the 55 secondary metabolites quantified in the leaves of *Foeniculum vulgare* Mill. (Table S3), 45 were statistically significant for at least one effect (accession, treatment, or their interaction). Of these, 7 (epicatechin gallate, polydatin, quercetin-3,4′-diglucoside, myricitrin, quercetin-3-glucuronide, isorhamnetin-3-O-rutinoside, and isorhamnetin) showed a significant accession × treatment interaction effect in the two-way ANOVA. Epicatechin and syringic acid, which showed residual heteroscedasticity, were instead analyzed using the Kruskal–Wallis test for treatment and accession effects separately (Section 4.6; Table S4); both showed a significant accession effect (epicatechin: *p* = 0.0012; syringic acid: *p* = 0.0024), while only epicatechin also showed a significant treatment effect (*p* = 0.0109). Patterns of metabolite accumulation varied with accession and treatment. Drought treatment significantly reduced the concentrations of 17 compounds, predominantly glycosylated flavonoids and the hydroxycinnamic acid derivative chlorogenic acid, with the most pronounced decreases observed in kaempferol-3-O-glucuronide, quercetin-3-O-arabinoside, and daidzein-7-O-glucoside. Conversely, 16 compounds increased under drought, including free flavonoid aglycones (quercetin, kaempferol) and several hydroxycinnamic acids (caffeic acid, ferulic acid, sinapic acid, cinnamic acid methylester). The most abundant class of polyphenols was flavonoids, namely glycosides of quercetin and kaempferol, as well as daidzein and luteolin, while the most abundant individual polyphenolic compound was chlorogenic acid. Significant genetic variation was observed in most polyphenols, while drought treatment generally reduced their synthesis.

**Figure 7:**
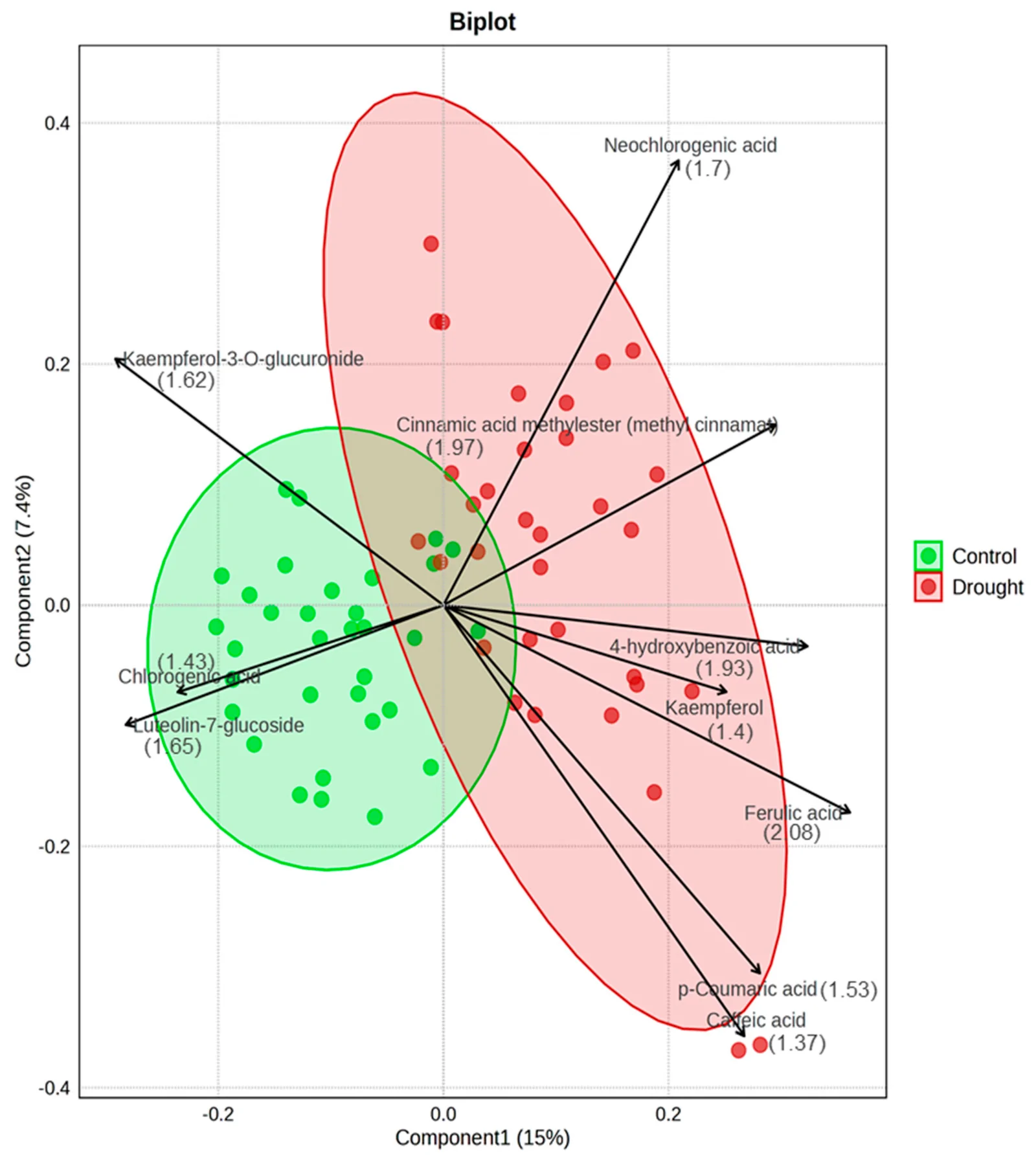
Partial Least Squares Discriminant Analysis (PLS-DA) biplot of secondary metabolite profiles in 11 *Foeniculum vulgare* accessions under control and drought treatment. The analysis was performed with MetaboAnalyst. Each point represents a sample, with green dots indicating control and red dots indicating drought-treated plants. The separation of groups along the components reflects differences in metabolite composition in response to drought stress. Numbers in parentheses represent Variable Importance in Projection (VIP) scores of each metabolite.

Partial Least Squares Discriminant Analysis (PLS-DA) of secondary metabolite profiles in eleven *Foeniculum vulgare* Mill. accessions revealed a clear separation between the control (green) and drought-treated group (red) (Figure 7). Component 1 and Component 2 explained 15.1% and 7.4% of the variance, respectively, with clustering reflecting differences in metabolite composition due to drought stress. The VIP scores from the PLS-DA model are shown in parentheses next to each metabolite. Metabolites with the highest VIP scores and strongly associated with the drought group included ferulic acid, caffeic acid, p-coumaric acid, kaempferol, 4-hydroxybenzoic acid, cinnamic acid methylester, and neochlorogenic acid, all of which were elevated under drought stress. In contrast, chlorogenic acid, luteolin-7-glucoside, and kaempferol-3-O-glucuronide were strongly associated with the control group, indicating their reduction under drought conditions.

**Figure 8:**
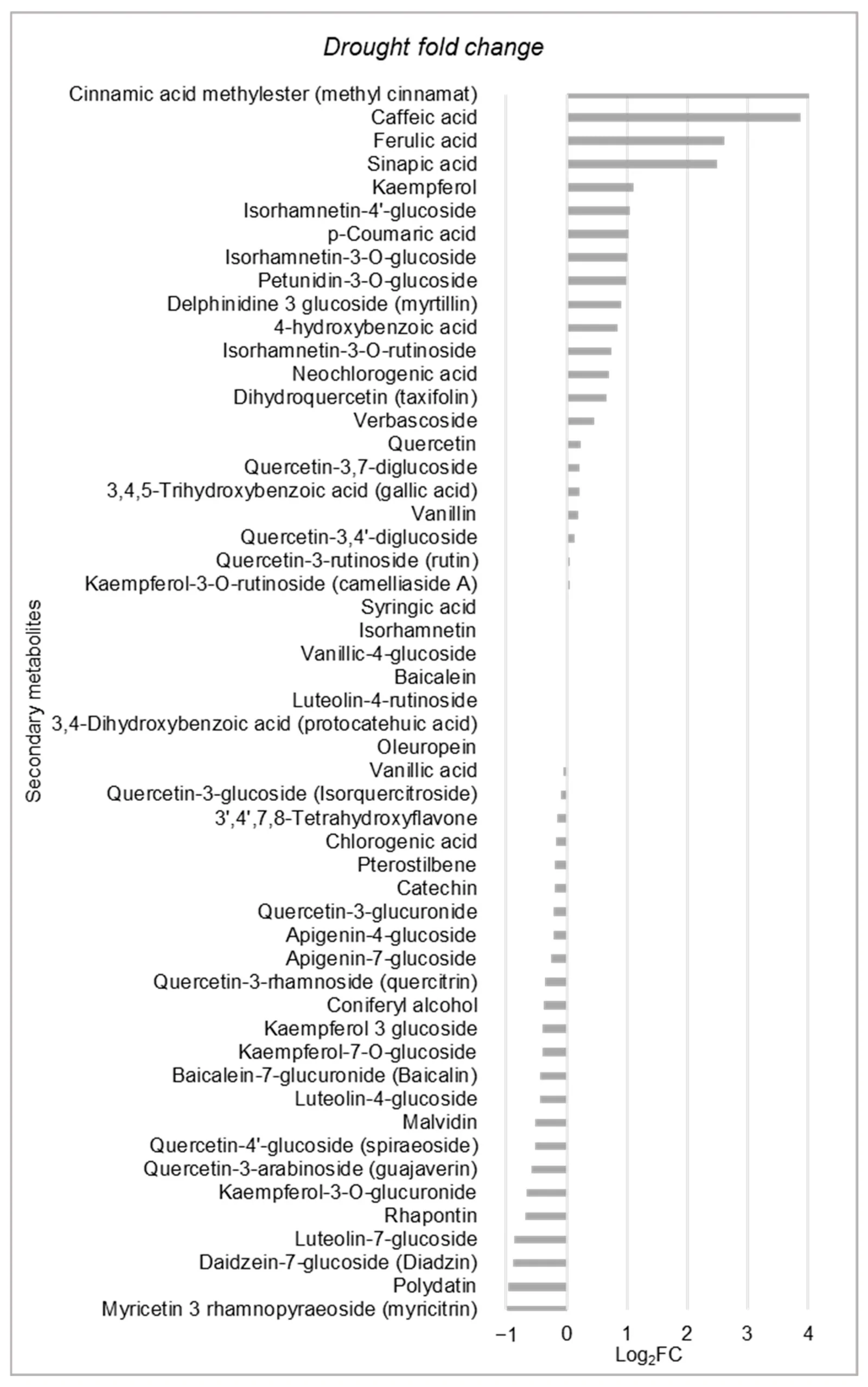
Drought-induced fold change in selected secondary metabolite concentrations in leaves of 11 *Foeniculum vulgare* Mill. accessions. Secondary metabolites were selected based on statistical significance of treatment effect (two-way ANOVA, post hoc Tukey, *p* < 0.05). Fold change was calculated as the log_2_ of the ratio of mean concentration under drought to mean concentration under control for each metabolite. Values > 0 indicate an increase under drought, while values < 0 indicate a decrease.

Drought-induced fold changes in the concentrations of selected secondary metabolites in the leaves of eleven *Foeniculum vulgare* Mill. accessions are presented in Figure 8. The displayed metabolites were chosen based on the statistical significance of the treatment effect, as determined by two-way ANOVA and post hoc Tukey tests (*p* < 0.05). Fold changes were calculated as the log_2_ ratio of the mean metabolite concentrations under drought and under control. Values above zero indicate upregulation during drought, while values below zero indicate downregulation under drought. Notably, cinnamic acid methyl ester, caffeic acid, and ferulic acid showed the largest positive fold changes, reflecting strong accumulation under drought stress. Conversely, luteolin-7-glucoside, diadzin, polydatin, and myricetin-3-rhamnopyranoside exhibited the greatest reductions under drought, as indicated by negative log_2_ fold changes. The extent of drought-induced shifts varied considerably among secondary metabolites.

**Figure 9:**
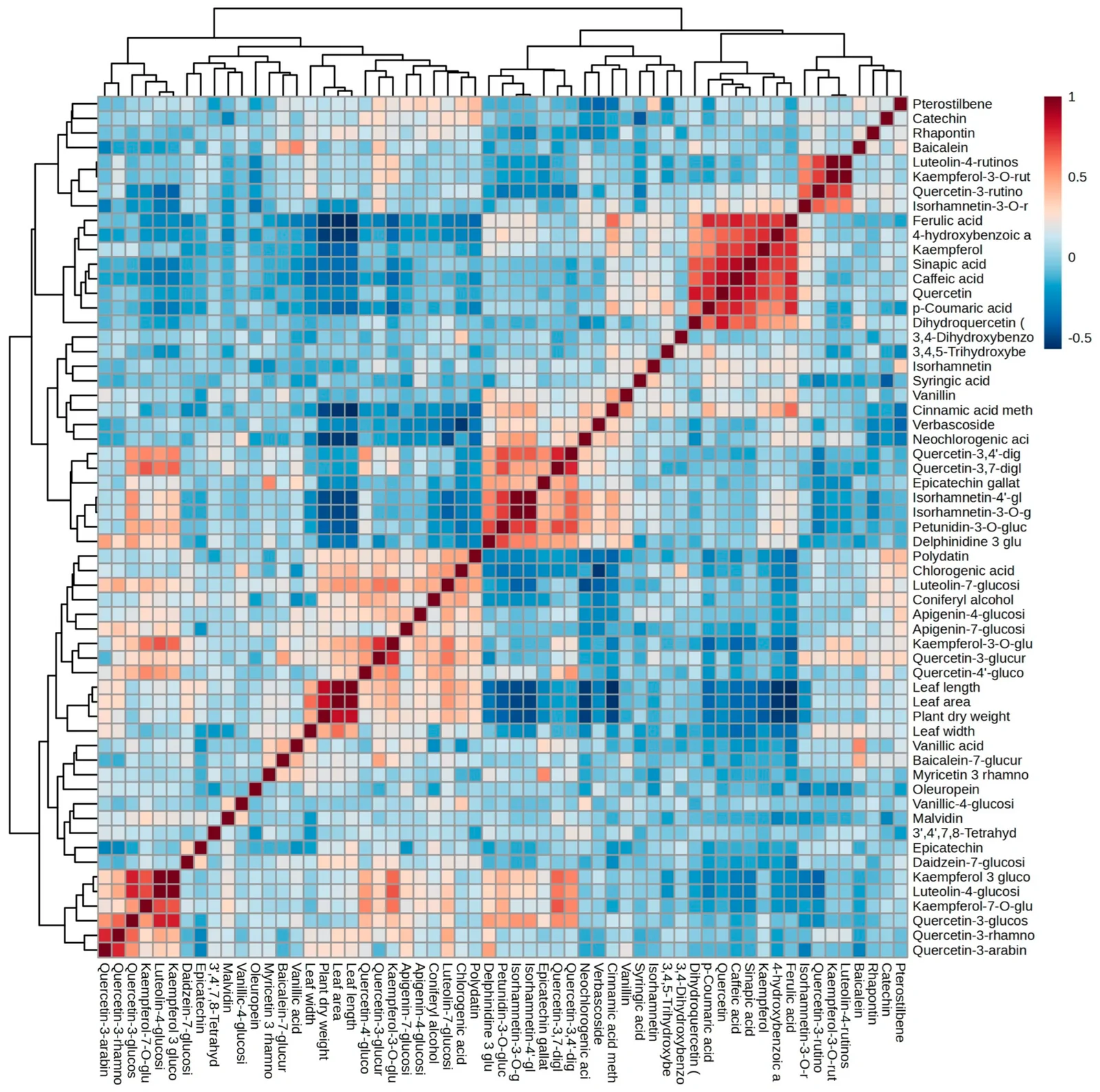
Heatmap of Pearson correlation coefficients between secondary metabolite abundances and morphological parameters across all *Foeniculum* accessions. Colors indicate the direction and strength of correlations, with hierarchical clustering applied to traits and metabolites. Correlation analysis was performed in MetaboAnalyst.

Figure 9 displays a heatmap of Pearson correlation coefficients between the abundances of secondary metabolites and key morphological parameters, namely, leaf area, leaf length, leaf width, and plant dry weight in leaves of all *Foeniculum vulgare* Mill. accessions. The correlation matrix reveals distinct clusters of secondary metabolites that covary with specific morphological measurements. All four morphological traits are clustered together and negatively correlated with the majority of secondary metabolites, including neochlorogenic acid, ferulic acid, and 4-hydrobenzoic acid. No strong positive correlations were observed between the morphological features and the majority of secondary metabolites. The clustering structure groups together metabolites with similar correlation profiles to morphological traits into two main clusters, providing insight into the metabolic modules closely associated with morphological diversity in fennel.

## 3. Discussion

The significant reduction in plant DW in response to drought across all *Foeniculum vulgare* Mill. accessions emphasizes the severity of the imposed stress and confirms that drought adversely affects biomass accumulation in fennel [14]. The observed accession-specific differences suggest genetic variability in drought tolerance, as evidenced by the smallest reduction in plant DW in IPT565 (1.5-fold) and the largest reduction in IPT574 (3.12-fold). Under drought conditions, many species exhibit a decrease in DM due to reduced stomatal conductance, which limits carbon assimilation necessary for photosynthesis [15]. While accession-specific differences were less pronounced for DM than for DW, the accession and treatment effects were statistically significant for stem DM and total DM, suggesting accession-dependent variation in drought resilience. A significant treatment effect was observed for leaf, stem, and total DM, all of which increased under water deficit conditions. This likely reflects a reduction in tissue water content rather than enhanced biomass accumulation, consistent with reports that drought-stressed plants generally retain proportionally less water in their tissues [16].

Fennel is generally considered a drought-sensitive species, although both drought-sensitive and drought-tolerant genotypes exist [11]. In the study by Parmoon et al. [14] reduced water availability during vegetative growth caused significant decreases in shoot biomass. A similar pattern was observed in the current study, as both leaf and stem DW, and consequently total DW, significantly decreased due to exposure to water deficit conditions. Patterns of decreasing vegetative growth were also evident through the reductions in leaf area, length, and width across all *Foeniculum vulgare* Mill. accessions following drought stress, which could indicate both direct and adaptive responses to water limitation [17]. Leaf area is one of the indicators of plant efficiency in resource utilization, especially under stress conditions such as drought. During drought, plants often reduce their leaf area to conserve water, signaling reduced efficiency in resource use and potential decline in plant productivity [18].

The substantial decrease in leaf area, especially in accessions IPT563, IPT567, and IPT574 (>4.7-fold), highlights the impact of drought on the photosynthetic surface, which could influence overall plant productivity [19]. Conversely, accessions such as IPT572, which showed the smallest reductions in leaf area and leaf length, appear to possess traits conferring a degree of drought tolerance. Smaller reductions in leaf morphology are generally interpreted as reflecting mechanisms that help preserve leaf structure or function [20]. Leaf width showed comparatively smaller changes under drought than the other leaf parameters, suggesting it is less responsive to water deficit. This relative stability may reflect an anatomical constraint on leaf width in fennel or a regulatory mechanism that maintains leaf integrity under water scarcity [17].

Plant growth and development are regulated by a chemically and structurally diverse group of hormones that are inevitably influenced by environmental conditions [21]. Indole-3-acetic acid (IAA) and abscisic acid (ABA) are key plant hormones that play crucial roles in regulating plant growth and development, as well as physiological processes, including responses to environmental stimuli, such as drought [22,23,24]. Under drought stress, changes in IAA content may reflect stress-induced growth remodeling, as auxin signaling interacts with ABA-mediated drought responses [25]. Similarly, during abiotic stress, particularly drought, plants exhibit a notable increase in ABA levels [23,24]. The increase in ABA is a central component of plant drought response, as ABA regulates stress-responsive gene expression and promotes stomatal closure to reduce water loss through transpiration [26,27]. Under water-deficit conditions, osmotic stress triggers ABA biosynthesis and signaling, leading to guard-cell responses that help conserve water and improve plant adaptation to drought [28]. The reduction in morphological traits under drought may be partly linked to hormonal reprogramming, particularly the accumulation of ABA and IAA. Increased ABA content is closely associated with drought signaling and stomatal regulation, whereas elevated or altered auxin levels may contribute to growth inhibition and developmental remodeling under stress conditions [29,30]. The negative correlations of both ABA and IAA with morphological parameters, together with their positive mutual correlation (Figure 6), could point toward coordinated ABA–auxin involvement in drought-induced growth adjustment in fennel plants. Beyond their immediate physiological effects, such coordinated hormonal shifts are also relevant from a breeding perspective: recent work has highlighted that crop drought resistance is governed not only by hormonal signaling but by its integration with transcriptional, post-translational, and epigenetic regulatory layers, and that a limited understanding of this multilevel regulation remains a major bottleneck in translating candidate drought-resistance genes into breeding programs [31]. The accession-dependent morphological responses observed here, particularly the reduced leaf area in IPT572, suggest that Croatian wild fennel accessions may harbor useful natural variation at one or more of these regulatory levels, warranting further molecular characterization.

Taken together, these hormonal and metabolic shifts could point to a coordinated physiological sequence rather than independent stress responses. The drought-induced increase in ABA content, alongside its established role in promoting stomatal closure [29], is broadly consistent with the reduced photosynthetic carbon assimilation associated with drought-induced stomatal limitation reported in other species [15] offering a plausible mechanistic link to the downstream suppression of TCA cycle intermediates observed in this study. The depletion of succinic acid, citric acid, and 2-ketoglutaric acid, the latter serving as the carbon skeleton for glutamate and glutamine synthesis [32] suggest that, as carbon availability becomes constrained, metabolic flux may be redirected from respiratory maintenance toward nitrogen assimilation and amino acid biosynthesis, consistent with the enrichment of the Alanine, aspartate, and glutamate metabolism and Arginine biosynthesis pathways [33,34]. The concurrent accumulation of GABA further supports activation of the GABA shunt, possibly as a compensatory route sustaining partial TCA-cycle flux under these conditions [35,36]. This ABA-driven changes in primary metabolism appears to operate alongside, rather than independently of, jasmonate-mediated regulation of secondary metabolism: methyl jasmonate (MeJA) is known to coordinate secondary with primary metabolism, including phenylpropanoid pathway activity [37] and its accumulation under drought may contribute to the observed shift toward free hydroxycinnamic acids and flavonoid aglycones, compounds associated with enhanced antioxidant capacity [38,39]. Overall, this suggests that ABA-mediated stomatal and metabolic restriction and MeJA-mediated activation of phenolic defense may operate as complementary, hormonally coordinated branches of the drought response in fennel, linking early physiological restriction of carbon assimilation to the later biochemical investment in oxidative stress protection. Mechanistically, this coordination between hormonal signaling and metabolic reprogramming is consistent with the proposed role of stress-responsive transcription factor families, such as NAC, MYB, WRKY, bHLH, and ERF/DREB, as convergence nodes that integrate ABA, jasmonate, and other hormonal inputs into unified transcriptional and metabolic outputs, allowing plants to coordinate responses to multiple, potentially co-occurring, stresses [40]. The parallel accumulation of ABA and MeJA observed in this study, alongside their apparent downstream effects on both primary carbon/nitrogen metabolism and phenylpropanoid biosynthesis, is consistent with such a convergence model [40]. However, the specific transcription factors mediating this crosstalk in fennel remain to be identified.

To mitigate the effects of drought stress at the biochemical level, plants have evolved adaptive mechanisms, including the accumulation of metabolites with diverse protective functions [41]. The accumulation or depletion patterns of specific metabolites may serve as biochemical indicators of drought stress intensity and associated oxidative damage [42]. In the present study, drought stress significantly shifted the glutathione pool toward an oxidized state, as evidenced by the marked increase in GSSG and the corresponding decline in the GSH/GSSG ratio, indicating the onset of oxidative stress across all fennel accessions. However, the significant accession × treatment interaction revealed genotypic variation in redox buffering capacity, with accession IPT563 having a notably higher GSH/GSSG ratio under control conditions, suggesting a more efficient antioxidant response that may contribute to greater drought tolerance [43]. Meanwhile, the significant increase in methionine sulfoxide content across all accessions under drought suggests heightened oxidative stress and the activation of protective mechanisms, as this metabolite is known to be involved in scavenging reactive oxygen species [44,45].

The predominance of positive fold change values in a comprehensive overview of drought-induced changes in the content of primary metabolites in *Foeniculum vulgare* Mill. leaves indicates that drought stress triggers a notable accumulation of many amino acids, which can act as osmoprotectants [3,46]. In the present study, the highest increase in content was observed for asparagine, indole-3-acetic acid, allantoin, arginine, and proline, which could suggest that these compounds have pivotal roles in the plant’s adaptive response, potentially through mechanisms related to nitrogen remobilization, growth regulation, osmoprotection, and antioxidant defense [47]. The observed accumulation of proline and abscisic acid, both well-established markers of stress response, as well as their strong positive correlation, further supports their function in osmotic regulation and signaling pathways essential for drought tolerance [4,29]. The marked decrease in 2-ketoglutaric acid is particularly relevant because this metabolite links central carbon metabolism with nitrogen assimilation by providing the carbon skeleton required for glutamate and glutamine metabolism [32].

The pathway analysis of drought-responsive primary metabolites in *Foeniculum vulgare* Mill. reveals significant metabolic adjustment, particularly in amino acid metabolism and central carbon metabolism. The treatment perturbs Alanine, aspartate and glutamate metabolism, which have previously been reported as significantly affected by drought in sesame (*Sesamum indicum*) [33] and oil palm (*Elaeis guineensis*) [34]. The strong enrichment of the Alanine, aspartate, and glutamate metabolism pathway, indicated by its low FDR value and high impact score, highlights the central role of amino acid turnover in the fennel’s drought response. The notable involvement of Arginine biosynthesis and Glyoxylate and dicarboxylate metabolism further suggests a coordinated shift in nitrogen and energy pathways, likely aiding in improved stress resilience and recovery [34]. Since there is no specific pathway library for *Foeniculum vulgare* Mill., the *Arabidopsis thaliana* pathway library was used for this analysis. While this approach highlights shared core metabolic processes among plants, it is important to note that species-specific pathways in fennel may not be completely represented. This limitation could affect the accuracy of the results. Depletion of 2-ketoglutaric acid, together with the enrichment of Alanine, aspartate and glutamate metabolism and Arginine biosynthesis, may reflect the redirection of available carbon skeletons toward amino acid biosynthesis and stress-protective nitrogen metabolism rather than maintenance of normal respiratory flux [48].

Furthermore, the accumulation of GABA may support the possible involvement of the GABA shunt as a compensatory route under drought stress [35]. In this pathway, glutamate-derived GABA is converted to succinate, thereby bypassing part of the TCA cycle and potentially sustaining downstream respiratory metabolism when conventional TCA cycle flux is constrained [36]. In the present study, the positive fold changes in GABA, glutamate, and alanine, together with the depletion of citric acid, succinic acid, and 2-ketoglutaric acid, suggest drought-induced reorganization of carbon and nitrogen metabolism [49]. Although succinic acid decreased under drought, this does not exclude GABA-shunt involvement, as succinate produced through this route may be rapidly consumed downstream or remain depleted due to the overall suppression of TCA-cycle activity. Therefore, fennel plants may partly maintain carbon flow and energy metabolism through GABA-shunt activity while redirecting nitrogen metabolism toward stress-protective amino acids.

The Partial Least Squares Discriminant Analysis (PLS-DA) clearly highlights the metabolic changes caused by drought stress in *Foeniculum vulgare* Mill., as shown by the distinct clustering of control and drought-treated samples. The significant variance explained by the first two components emphasizes the strong effect of water availability on primary metabolite profiles. This separation confirms that drought triggers major changes in metabolic processes, reflecting physiological adjustments to cope with stress. The identification of key metabolites with high VIP scores, such as dimethylglycine, choline, phenylalanine, and glutamine, offers insight into specific biochemical pathways involved in drought adaptation. These metabolites play roles in osmoprotection, membrane stability, and nitrogen metabolism, which are essential under water-deficient conditions [50,51,52,53,54]. In addition, numerous primary metabolites can also serve as precursors of other downstream compounds, such as phenylalanine, which serves as a precursor of polyphenolics, bioactive compounds generated via phenylpropanoid and flavonoid pathways, thus linking primary and secondary metabolism [55].

The link between primary and secondary metabolism under drought stress could be further supported by changes in stress-related signaling molecules, particularly MeJA. As an active jasmonate derivative, MeJA is involved in the regulation of stress-responsive genes and contributes to plant adaptation under adverse environmental conditions [56]. Under drought conditions, increased MeJA content may promote protective responses such as stomatal regulation and enhanced antioxidant capacity, thereby limiting water loss and oxidative damage [56,57]. Importantly, jasmonate signaling is also closely associated with the regulation of phenylpropanoid metabolism, suggesting that MeJA accumulation may contribute to the observed changes in polyphenolic compounds in drought-treated fennel plants [37].

Polyphenolic compounds are important components of plant stress responses due to their contribution towards ROS scavenging and photoprotection under adverse environmental conditions [58,59,60]. In the present study, the drought-induced accumulation of several hydroxycinnamic acids and free flavonoid aglycones suggests activation of phenylpropanoid-related defense pathways [38,39], whereas the reduction in several glycosylated flavonoids may indicate a shift in the balance between the storage forms and phenolic compounds more directly involved in stress-related redox regulation and antioxidant defense [61].

The PLS-DA of secondary metabolites in *Foeniculum vulgare* Mill. effectively differentiates between control and drought-treated plants, thereby confirming that drought induces significant changes in secondary metabolite composition. The distinct clustering indicates that the drought response involves notable modulation of secondary metabolites. The higher VIP scores for ferulic acid, cinnamic acid methylester, 4-hydroxybenzoic acid, and neochlorogenic acid highlight the importance of phenolic and related compounds in drought adaptation. As these metabolites are widely recognized for their roles in reducing oxidative damage caused by drought-induced stress [39], their prominence in distinguishing treatment groups emphasizes the biochemical shifts that occur beyond primary metabolism, suggesting that secondary metabolites play a significant role in the protective and adaptive mechanisms activated in fennel under drought. The observed fold changes in secondary metabolite concentrations offer a detailed insight into the complex and selective metabolic adjustments. Cinnamic acid methylester, caffeic acid, and ferulic acid showed the largest positive fold changes, identifying free hydroxycinnamic acids as the compounds most strongly favored under drought, which likely support the plant’s antioxidant defense during water-deficit conditions. Conversely, the significant decreases in luteolin-7-glucoside and daidzin indicate a targeted downregulation or redirection of specific flavonoid derivatives during drought. The observed accumulation of free flavonoid aglycones and hydroxycinnamic acids, alongside a concurrent decline in their glycosylated counterparts, indicates a shift in the balance between conjugated and free phenolic forms under drought. Two mechanisms could produce this pattern: hydrolysis of pre-existing glycoside pools, or preferential accumulation of newly synthesized aglycones without subsequent glycosylation. These alternatives cannot be distinguished from single-timepoint concentration data. This metabolic shift may contribute to the antioxidant response accompanying the observed decline in the GSH/GSSG ratio, as free aglycones generally exhibit stronger in vitro radical-scavenging activity than their glycosylated forms [62]. This differential regulation emphasizes the metabolic flexibility of fennel, where the balance between metabolite synthesis and degradation is carefully adjusted in response to environmental stress.

While most secondary metabolites responded consistently across accessions, six compounds: epicatechin gallate, polydatin, quercetin-3,4′-diglucoside, myricitrin, quercetin-3-glucuronide, and isorhamnetin showed a significant accession × treatment interaction, indicating that the direction or magnitude of their drought response was genotype-dependent. Notably, these are predominantly flavonoid conjugates and flavan-3-ols rather than the free hydroxycinnamic acids that dominated the overall drought response. This suggests that the core phenylpropanoid response to drought is broadly conserved among Croatian wild fennel accessions, whereas variation between genotypes is concentrated in the conjugated and stored fraction of the polyphenol pool. Such genotype-specific modulation of storage forms parallels the interaction observed for the GSH/GSSG ratio and identifies the conjugated flavonoid pool as the more variable component of the drought response and therefore the more promising target for accession-level selection.

Furthermore, the correlation analysis revealed a predominantly negative association between polyphenolic compounds and morphological traits, including leaf area, leaf width, leaf length, and plant dry weight (Figure 9). However, several individual compounds deviated from this trend and showed positive correlations with one or more morphological parameters. This trend is consistent with a growth–defense trade-off, in which reduced leaf expansion is accompanied by enhanced accumulation of compounds involved in oxidative stress protection and stress adaptation [63].

Beyond their role in drought-response characterization, Croatian wild fennel accessions may represent a valuable genetic and phytochemical resource. *Foeniculum vulgare* Mill. is widely recognized for its culinary, medicinal, and aromatic value, largely associated with its diverse profile of volatile compounds, phenolic acids, flavonoids, and other bioactive constituents [1], and wild fennel has been reported to exhibit higher antioxidant potential and total phenolic and flavonoid contents than some medicinal and edible types [64]. Against this background, the accession-dependent differences observed here suggest that wild populations from the Croatian coast harbor distinct adaptive and biochemical traits. The genotype-specific responses in leaf area and length indicate that selection for drought resilience in fennel should prioritize accessions that maintain leaf surface area under water deficit. In contrast, metabolite-based clustering of accessions and compounds with high VIP scores offers candidate biomarkers for marker-assisted selection. Since hormonal and metabolic responses to drought are ultimately triggered by upstream stress perception at the cell surface, future studies could also examine the role of CrRLK1L (Catharanthus roseus RLK1-like) receptor-like kinases in fennel, a protein family recently shown to integrate diverse abiotic and biotic stress signals, including cell wall integrity sensing with downstream hormonal and developmental responses [65]. Characterizing such early signaling components could help clarify how the ABA- and MeJA-associated changes described here are initiated at the cellular level, and whether they are shared across drought-tolerant and drought-sensitive fennel accessions. Accessions combining favorable morphological performance with the accumulation of stress-protective and bioactive metabolites would therefore be of particular interest for conservation and breeding, positioning this material not only as experimental germplasm for drought-stress assessment but as a regionally adapted resource for developing resilient fennel genotypes with desirable phytochemical profiles.

## 4. Materials and Methods

### 4.1. Plant Material

Fennel seeds were collected from 11 accessions originating from various locations across the Istra and Kvarner region in Croatia. The accessions were designated with internal labels (IPT codes) corresponding to their geographic origins as follows: IPT562 (Barban), IPT563 (Raša), IPT564 (Valmade), IPT565 (Plomin), IPT566 (Vodnjan), IPT567 (Krk), IPT568 (Rijeka), IPT569 (Split), IPT570 (Kamenjak), IPT572 (Vodnjan), and IPT574 (Pula) (Table S5).

### 4.2. Experimental Design

The experiment was set up in a non-heated greenhouse at the Institute of Agriculture and Tourism in Poreč, Croatia (45°13′20.351″ N, 13°36′6.397″ E). Fennel seeds were planted in polyethylene pots containing a 3:1 mixture of substrate (Potgrond H, Klasmann, Geeste, Germany) and perlite (Agroperl, Stauss-Perlite, Pölten, Germany). The experiment included 11 fennel accessions. One-month-old plants were transferred to 0.4 L (10 × 7.7 × 5.5 cm) pots with the same soil mixture as previously described. Two-month-old plants were divided into two groups: watered control and non-watered drought group. The experiment was conducted for 15 days. In the control group, plants were watered daily to maintain optimal soil moisture. In the drought group, watering was withheld, and drought conditions were sustained until 60% of the plants in the group exhibited visible turgor loss. When this threshold was reached, plants were re-watered, marking the end of one drought cycle. This process of drought and recovery was repeated three times during the 15-day experimental period, resulting in a total of three drought cycles. At the end of the 15 days, all plants were sampled for further analysis. The morphological measurements were conducted on 330 plants, which included 11 accessions, 2 treatments, 5 plants, and 3 repetitions. Of the five plants per accession × treatment combination within each block, one plant per block was also used for metabolite analysis, resulting in three individually measured biological replicates (*n* = 3) per accession × treatment combination (66 samples total).

### 4.3. Dry Matter Content

The dry matter content (DM) was determined by drying the fennel leaf samples at 105 °C in a forced hot air circulation oven (Memmert UF160, Schwabach, Germany) until a consistent weight was achieved in all replicates. Before drying, fresh leaf samples were gently blotted with paper towels to remove excess surface moisture and then weighed to record their initial fresh weight. Samples were then spread evenly on trays and placed in an air-circulation oven. The samples were weighed at regular intervals until two consecutive measurements showed no further change, indicating that complete drying had been achieved. The dry matter content was calculated as the ratio of dry weight to fresh weight, expressed as a percentage. DM was determined separately for leaves and stems of each plant.

### 4.4. Leaf Area Determination

Leaf area was determined using a LI-3000C Portable Leaf Area Meter (LI-COR Biosciences, Lincoln, NE, USA). For each sample, individual leaves were carefully detached and immediately scanned following the manufacturer’s instructions. The LI-3000C utilizes narrow-band light-emitting diodes (LEDs) in conjunction with paired detectors within the scanning head, allowing for precise and non-destructive measurement of leaf area, as well as leaf length and width. Each detached leaf was passed through the scanning head from base to apex, and measurements were recorded for subsequent analysis.

### 4.5. Metabolite Analysis

A comprehensive panel of 79 primary and 57 secondary metabolites was quantified, encompassing multiple biochemical classes. Leaf samples from both drought-treated and control fennel plants were analyzed for metabolite profiles using high-performance liquid chromatography coupled with tandem mass spectrometry (HPLC-MS/MS), following a previously established protocol [66]. Briefly, metabolites were extracted by homogenizing 30 mg of freeze-dried and finely powdered leaf tissue in 1.5 mL of 80% methanol. The resulting extracts were thoroughly mixed and then filtered through 0.22 µm membrane filters to remove particulates before injection. Chromatographic separation was performed using a reversed-phase ultra-performance liquid chromatography (RP-UPLC) system equipped with two high-precision solvent delivery units (Shimadzu Nexera LC-40DX3, Kyoto, Japan), an autosampler (Shimadzu Nexera SIL-40CX3, Kyoto, Japan), and a column compartment (Shimadzu Nexera CTO-40C, Kyoto, Japan), ensuring consistent sample injection and optimal temperature control. Separated metabolites were subsequently detected and quantified using a triple quadrupole (QqQ) mass spectrometer (Shimadzu LCMS8045, Kyoto, Japan) operating in electrospray ionization (ESI) mode. Following the methodology established by Major et al. [67], chromatographic separation of secondary metabolites was achieved by injecting a 1 μL sample on a C18 core–shell column 150 mm × 2.1 mm, 2.7 μm (Agilent, Palo Alto, CA, USA) maintained at 37 °C. The mobile phase system consists of water with 0.1% acetic acid (A) and methanol with 0.1% acetic acid (B). The flow rate was set to 0.35 mL/min with the following gradient profile: 0–0.75 min: 95% A0.75–15 min: 95% to 50% A15–15.1 min: 50% to 0% A15.1–20 min: 0% A20–20.1 min: 0% to 95% A20.1–25 min: 95% A (re-equilibration). For primary metabolite analysis chromatographic separation was performed on a Discovery^®^ HS F5-3 column (2.1 mm × 150 mm, 3 μm core–shell, Sigma-Aldrich, St. Louis, MO, USA) maintained at 37 °C, by injecting 1 μL of the sample using a linear gradient between mobile phase A (water with 0.1% formic acid) and mobile phase B (methanol with 0.1% formic acid), at a flow rate of 0.25 mL/min. The gradient profile was as follows: 0–2 min, 100% A; 2–15 min, gradient from 100% A to 5% A; 15–20 min, 5% A; 20–20.1 min, return to 100% A; and 20.1–25 min, 100% A. Target compounds were identified and quantified by matching retention times, characteristic precursor/product ion transitions, and peak areas to those of authentic standards. The ratio of reduced glutathione to oxidized glutathione was calculated by dividing the concentration of GSH by the concentration of GSSG.

### 4.6. Statistical Analysis

The metabolic data were analyzed using analysis of variance (ANOVA) with Statistica 13.4 (TIBCO, Inc., Palo Alto, CA, USA). Significant differences were identified at *p* ≤ 0.05, and the means of homogeneous groups were compared using Tukey’s post hoc test. Shapiro–Wilk normality tests and Levene’s test for homogeneity of variances were performed for all metabolites. Two metabolites (epicatechin, syringic acid) remained heteroscedastic and were therefore analyzed using the non-parametric Kruskal–Wallis test instead of ANOVA, followed by Conover–Iman post hoc pairwise comparisons with Bonferroni-adjusted *p*-values. Exact F-values, numerator/denominator degrees of freedom, and partial η^2^ effect sizes are also reported in Table S4.

A multivariate approach was used to identify the metabolites contributing most to group separation between control and drought treatments. Partial Least Squares Discriminant Analysis (PLS-DA) was performed separately for primary and secondary metabolite datasets using Statistica 13.4 (TIBCO, Inc., Palo Alto, CA, USA). Model validity was assessed using 5-fold cross-validation combined with 1000 permutation tests, and model performance was evaluated based on R^2^ (goodness of fit) and Q^2^ (predictive ability) values. The number of latent components retained for each model was selected based on the component number that maximized Q^2^ while avoiding overfitting. The resulting R^2^Y/Q^2^ values were: R^2^Y = 0.8251, Q^2^ = 0.7745 for primary metabolites, and R^2^Y = 0.7081, Q^2^ = 0.5469 for secondary metabolites. Permutation testing confirmed that the separation between groups was not attributable to chance (*p* = 0.001; Supplementary Table S4). Variable Importance in Projection (VIP) scores were used to identify the metabolites contributing most strongly to group discrimination. Regarding the Pearson correlation matrices presented in Figure 6 and Figure 9, these were generated using the Correlation Analysis module in MetaboAnalyst 6.0 (Auto Scaling) [68].

## Figures and Tables

**Table 1:** Leaf dry weight (DW), dry matter (DM), and morphological traits of 11 *Foeniculum vulgare* accessions under control and drought treatment. Statistical significance of the main effects of accession (Ac), treatment (Tr), and the interaction (Ac × Tr) was assessed using a two-way ANOVA. Statistically significant *p*-values (<0.05) are formatted in bold.

	Leaf DW (g)	Stem DW (g)	Total DW (g)	Leaf DM (%)	Stem DM (%)	Total DM (%)	Leaf Area (cm^2^)	Leaf Length (mm)	Leaf Width (cm)
Accession (Acc)	**0.000**	**0.000**	**0.000**	0.489	**0.000**	**0.016**	**0.000**	**0.000**	**0.000**
Treatment (Tr)	**0.000**	**0.000**	**0.000**	**0.000**	**0.000**	**0.000**	**0.000**	**0.000**	**0.000**
Acc × Tr	**0.000**	**0.015**	**0.000**	0.361	0.325	0.080	**0.000**	**0.000**	0.072

**Table 2:** Parameters with significant main effects of accession and treatment and a non-significant interaction effect. Leaf width and dry matter (DM) of *Foeniculum vulgare* Mill. accessions under control and drought treatments are shown separately for leaves and stems and as their sum. Data are presented as mean ± standard error for each accession and treatment (*n* = 5). Different lowercase letters indicate statistically significant differences between mean values at *p* < 0.05 determined by a two-way ANOVA and Tukey’s test.

	Leaf DW (g)	Stem DW (g)	Total DW (g)	Leaf DM (%)
Accession				
IPT562	18.90 ± 0.73	19.65 ± 0.83 a–c	19.25 ± 0.65 ab	0.97 ± 0.04 b–f
IPT563	20.05 ± 0.46	19.44 ± 0.80 bc	19.81 ± 0.53 ab	1.10 ± 0.03 ab
IPT564	19.04 ± 0.61	18.88 ± 0.65 c	19.07 ± 0.57 ab	1.15 ± 0.04 a
IPT565	18.82 ± 0.65	19.67 ± 1.37 a–c	18.73 ± 0.86 b	1.03 ± 0.02 a–d
IPT566	19.08 ± 0.53	21.11 ± 0.59 a–c	19.58 ± 0.53 ab	0.94 ± 0.03 c–f
IPT567	20.40 ± 0.95	19.65 ± 0.72 a–c	20.26 ± 0.74 ab	1.08 ± 0.03 a–c
IPT568	19.03 ± 0.71	19.47 ± 1.06 bc	19.15 ± 0.70 ab	0.99 ± 0.03 b–e
IPT569	19.71 ± 0.72	23.32 ± 1.01 a	21.23 ± 0.87 a	0.82 ± 0.04 f
IPT570	19.83 ± 0.56	23.17 ± 0.69 ab	21.13 ± 0.64 ab	0.86 ± 0.03 ef
IPT572	19.70 ± 0.39	20.25 ± 0.72 a–c	19.83 ± 0.45 ab	0.90 ± 0.03 d–f
IPT574	19.47 ± 0.76	20.80 ± 1.07 a–c	19.99 ± 0.80 ab	1.10 ± 0.05 ab
Treatment				
Control	17.46 ± 0.16 b	18.63 ± 0.29 b	17.74 ± 0.16 b	1.05 ± 0.02 a
Drought	21.48 ± 0.29 a	22.36 ± 0.42 a	21.92 ± 0.30 a	0.94 ± 0.02 b

## Data Availability

Dataset available on request from the authors. Data for primary and secondary metabolites can be found here https://zenodo.org/records/21903857 (accessed on 12 August 2026.), https://doi.org/10.5281/zenodo.21903857.

## Supplementary Materials

The following supporting information can be downloaded at https://www.mdpi.com/article/10.3390/plants15172652/s1, Figure S1. Alanine, aspartate, and glutamate metabolic pathway. Nodes represent specific metabolites with those detected as significantly changed in the present study formatted in bold; Figure S2. Representative images of Foeniculum vulgare Mill. plants under (A) control and (B) drought treatment conditions; Figure S3. Relative water content (RWC) in leaves of 11 *Foeniculum vulgare* Mill. accessions under control and drought treatment; Table S1. Concentrations (mean ± standard error) of primary metabolites in leaves of eleven *Foeniculum vulgare* Mill. accessions under control and drought treatments, grouped by class, with different lowercase letters within a column indicating significant differences at *p* < 0.05 (two-way ANOVA, Tukey’s post hoc test) and main effects of accession (Acc), treatment (Tr), and their interaction (Acc × Tr) denoted ns, *, **, *** for *p* > 0.05, ≤0.05, ≤0.01, and ≤0.001, respectively; Table S2. Pathway analysis overview of primary metabolites significantly changed under drought conditions in *Foeniculum vulgare* accessions, generated with MetaboAnalyst. Primary metabolites were selected based on statistical significance of treatment effect (two-way ANOVA, *post hoc* Tukey, *p* < 0.05). The table includes the top 13 identified metabolic pathways, with False discovery rate (FDR) values < 0.03, indicating significant pathway enrichment under drought stress; Table S3. Concentrations (mean ± standard error) of secondary metabolites in leaves of eleven *Foeniculum vulgare* Mill. accessions under control and drought treatments, grouped by class, with different lowercase letters within a column indicating significant differences at *p* < 0.05 (two-way ANOVA, Tukey’s post hoc test) and main effects of accession (Acc), treatment (Tr), and their interaction (Acc × Tr) denoted ns, *, **, *** for *p* > 0.05, ≤0.05, ≤0.01, and ≤0.001, respectively; Table S4. Statistical analysis details; Table S5. Eleven *Foeniculum v.* accessions with their internal labels, geographic origins, and coordinates (latitude and longitude); Table S6. Discriminant primary and secondary metabolites (VIP > 1.5) identified by PLS-DA, with their reported biological functions and direction of change under drought stress.
